# Case Report: Metastatic right ventricular tumor and pulmonary embolism from cervical cancer post COVID-19 infection

**DOI:** 10.3389/fimmu.2026.1845280

**Published:** 2026-06-15

**Authors:** Yuemei Yang, Xiaohui Zhu, Danli Hu

**Affiliations:** 1 Department of Respiratory and Critical Care Medicine, The Fourth Affiliated Hospital of Nanjing Medical University, Nanjing, China; 2 Department of General Practice, Beijing Chao-yang Hospital, Capital Medical University, Beijing, China

**Keywords:** cervical cancer, COVID-19 infection, metastatic right ventricular tumor, prognosis, pulmonary embolism

## Abstract

The intracavitary cardiac metastasis from squamous cell carcinoma of the uterine cervix combined with pulmonary embolism is extremely rare, with mean survival less than six months. We report a case of a 52-year-old female with stage IVB cervical squamous cell carcinoma (SCC) and cardiac metastasis presented with exertional dyspnea and chest pain concerning pulmonary embolism (PE) post COVID-19 infection. After empirical anticoagulation and anti-infective therapy, the intracardiac mass and pulmonary embolism persisted. Subsequent PET-CT and histopathological examination revealed advanced cervical squamous cell carcinoma with suspected tumor thrombus involving the right heart and pulmonary artery. Given extensive metastatic disease and high bleeding risk, the multidisciplinary team discussed individualized systemic therapy and suggested conservative management. The patient survived 14 months after diagnosis.

## Introduction

Cervical cancer is the second most common malignancy among women worldwide (1). In its advanced stages, it often metastasizes to sites such as the lungs, brain, or supraclavicular lymph nodes; however, cardiac involvement is exceedingly rare (2). Autopsy data indicate that the incidence of cardiac metastasis is between 2.3% and 18.3% (3, 4).

Cancers in the upper part of the diaphragm, such as those in the lungs, breast, esophagus, and malignant lymphoma, are more likely to metastasize to the heart (4, 5). However, metastasis of infra-diaphragmatic organ malignancies to the heart is extremely rare. This may be related to the continuous movement of the myocardium, the rapid circulation of blood, and the outflow of lymph from the heart.

Although cardiac metastases are often asymptomatic and discovered only at autopsy, it can produce a wide range of clinical symptoms that depend on their size, anatomical location, and degree of tissue infiltration (4). There are four pathways for cardiac metastasis of malignancy, including regressive spread by lymph node, directly form adjacent viscera, blood circulation and vena cava. When a cervix cancer metastasizes to heart, it is more difficult to discover the fatal condition such as pulmonary embolism. Hence, due to the rarity of this condition, it lacked the standardized management. Here, we report a case of cervical carcinoma with metastasis to right ventricle combined with pulmonary emboli post COVID-19 infection.

## Results

A 53-year-old female presented to emergency room with low-grade fever, progressively worsening shortness of breath for 5 days, and associated right chest pain.

Her nucleic acid amplification tests for COVID-19 were positive. The patient did not receive anti-viral treatment like nirmatrelvir/ritonavir due to beyond the established optimal window (within 48 hours) and the scarce medical resources. The blood test for white cell, neutrophile, C-reactive protein and ESR were increased. The lung CT scan revealed right lower lobe pneumonia and possible pulmonary embolism. During this period, the anti-infection therapy of moxifloxacin for two days did not take effect at local hospital. It was empirical antibiotic therapy because neutrophilia, elevated inflammatory markers, and chest CT findings of right lower lobe pneumonia raised concern for bacterial co-infection. The computed tomography pulmonary angiography (CTPA) revealed the pulmonary embolism in the lower lobe of the right lung. The echocardiogram showed multiple low-echo mobile deposits in the tricuspid valve, right ventricle and right atrium, ranging from 0.9 cm to 3.2 cm. However, she had no any past history of cardiovascular and pulmonary disease. The patient underwent surgery for lower extremity varicose veins two years ago. She had 2 sons and one daughter and was currently in the perimenopausal period. She was primarily diagnosed as bacterial pneumonia combined with pulmonary embolism and right ventricular thrombus post COVID-19 infection. The patient was then admitted to the hospital and started on a treatment dose of unfractionated heparin (UFH) sustaining the APTT ranging from 60–80 seconds for 10 days.

This patient underwent a re-examination of echocardiography twice, and the results showed a consistent mass-like, medium-echo lesion in the right atrium and right ventricle, which could move with blood flow and involved the lateral tricuspid valve and chordae tendineae, approximately ranging from 1.9 to 4.9 centimeters. And the lesion was considered a space-occupying lesion.

**Figure 1:**
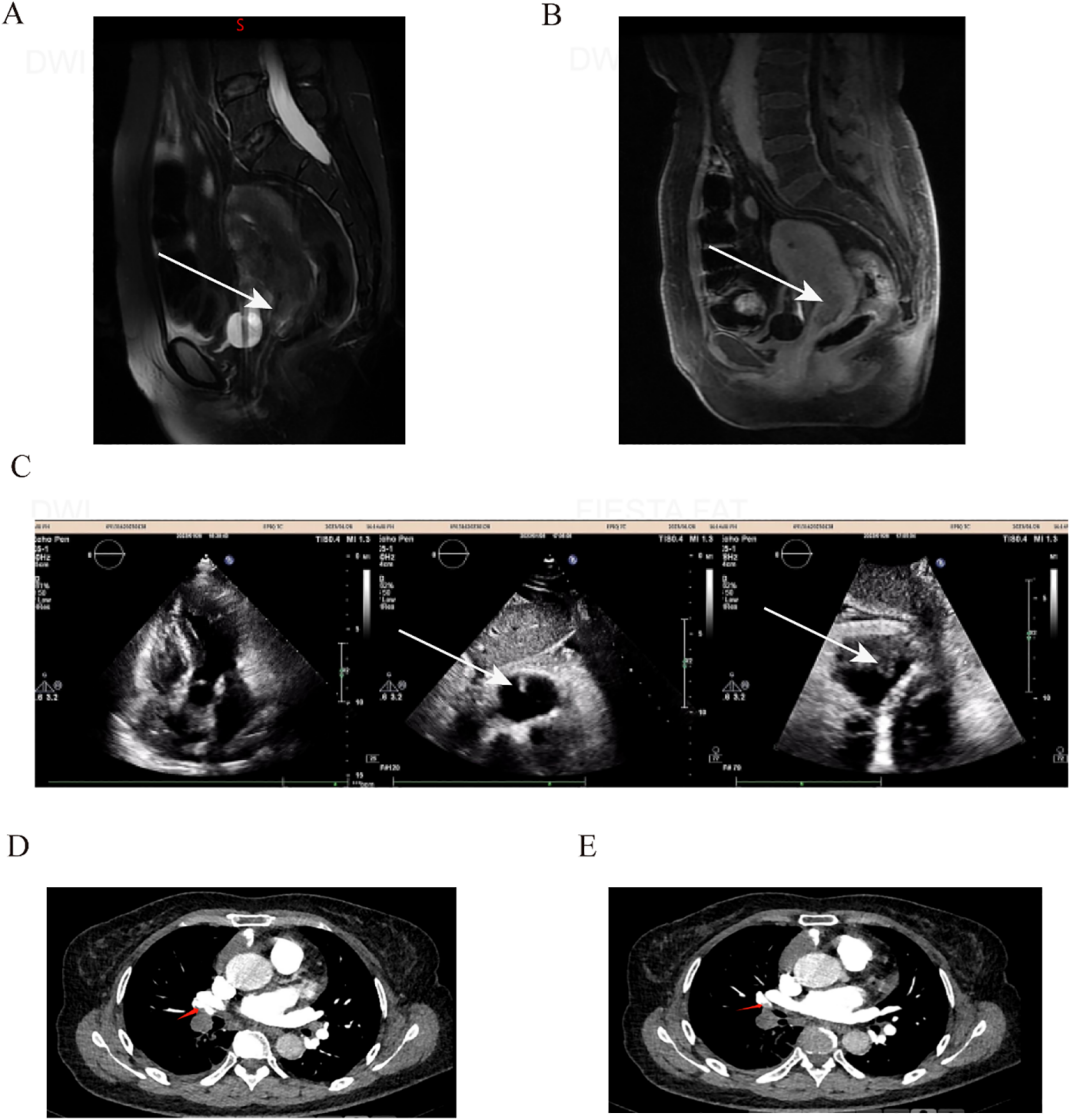
Magnetic resonance, echocardiography images and computed tomography pulmonary angiography. **Figure 1**. Magnetic Resonance and Echocardiography images. **(A, B)** T2 and Cor LAVA sequences of contrast-enhanced pelvic MRI showing the cervical lesion. **(C)** Transthoracic echocardiogram image. The white arrow indicated the lesions. **(D, E)** Computed tomography pulmonary angiography. The red arrow indicated a low-density filling defect in the pulmonary artery of the right lower lobe and its distal branches.

The blood tumor marker tests showed a significant increase in squamous cell carcinoma (SCC) and a slight increase in cytokeratin 19 fragment (CYFRA19), Carcinoembryonic antigen (CEA) and neuron-specific enolase (NSE). The chest CT scan did not discover primary malignancy. During the course of 10-day anticoagulation treatment with unfractionated heparin (UFH), the patient had an abnormal vagina bleeding. To further exclude the gynecological origin tumors, she was performed the transvaginal gynecological ultrasound examination. The result revealed a 3.1*2.7-centimeter hypoechoic mass in the lower uterine segment. The pelvic enhanced magnetic resonance imaging examination revealed high DWI signal approximately 3.3*3.0*1.8 centimeters adjacent to the cervix and in the upper 1/3 of the vagina, suggesting a possibility of cervix cancer (**Figure 1**). There were several small lymph nodes in the right parametrium, approximately 3 to 7 millimeters, which were highly suspicious of lymph node metastasis. The rechecked CTPA revealed consistent pulmonary embolism in the lower lobe of the right lung (**Figure 1**). Then the patient underwent positron emission tomography – computed tomography (PETCT) scan, and the result revealed that the cervix was thickened and had an irregular shape, indicating a high possibility of malignancy. Multiple lymph nodes of varying sizes with elevated metabolism were found in the supraclavicular area, mediastinum, bilateral hilar regions, para-aortic area, bilateral iliac vascular distribution areas, and the left inguinal region, which highly indicated metastasis. Metabolic active foci were also observed in the right atrium, right ventricle, and pulmonary artery of the lower lobe of the right lung, which highly indicated cancerous thrombi.

During the 10-day course of therapeutic anticoagulation with unfractionated heparin (UFH), the patient developed abnormal vaginal bleeding. UFH was temporarily withheld and a gynecology consultation was obtained. After bleeding was controlled, anticoagulation was cautiously resumed with close monitoring, balancing persistent PE and tumor-associated thrombosis against the risk of rebleeding.

**Figure 2:**
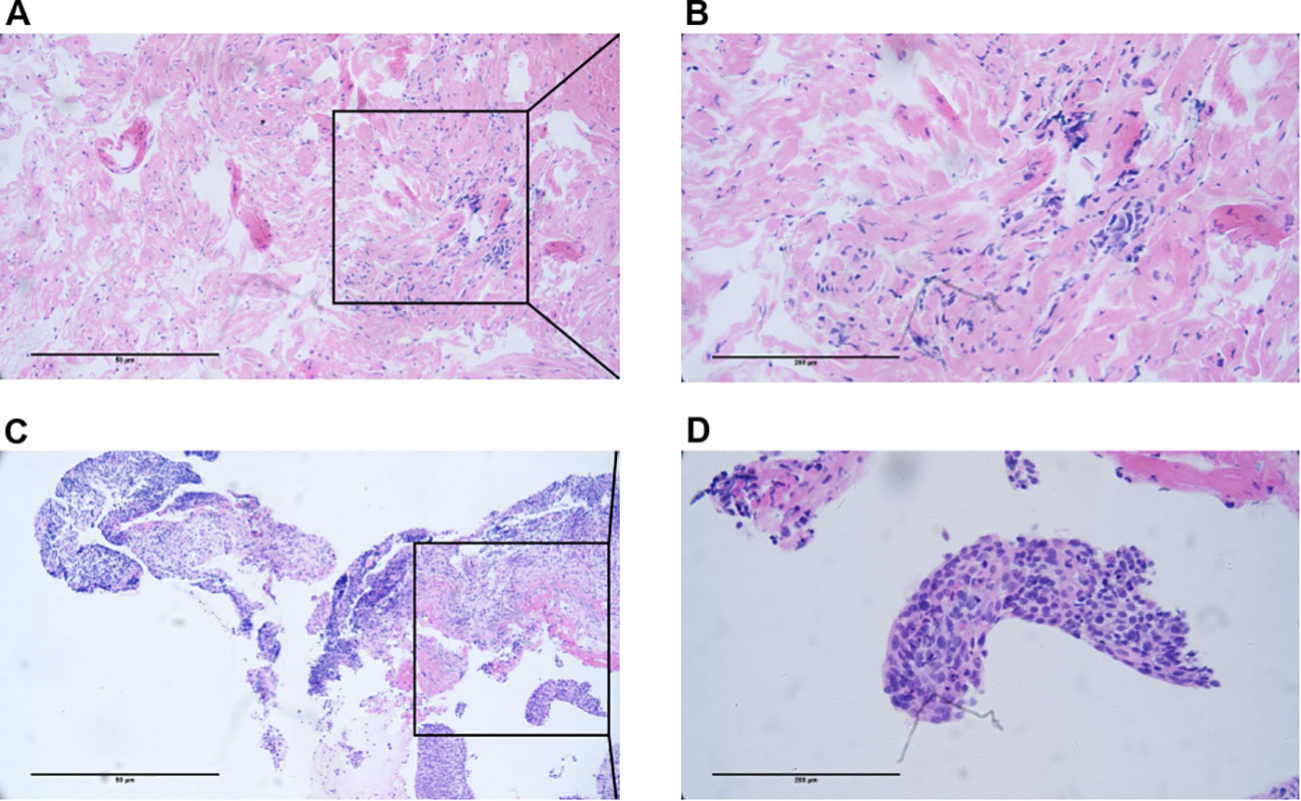
Pathological images of cervical cancer tissue biopsy. **Figure 2**. Hematoxylin and eosin staining (HE) of cervical mucosal biopsy. **(A)** Low-power view showing atypical squamous epithelial cells (black box). Scale bar = 50 μm. **(B)** High-power magnification of the boxed area in **(A)**, demonstrating the cytological features of the atypical squamous cells. Scale bar = 200 μm. **(C)** Low-power view showing fragmented and detached atypical squamous epithelial cells infiltrating the cervical stroma (black box). Scale bar = 50 μm. **(D)** High-power magnification of the boxed area in **(C)**, revealing stromal invasion by the atypical squamous epithelial cells. Scale bar = 200 μm.

Then the patient underwent gynecological TCT test. The result revealed cervical HPV-16 with a viral load of 10^6 copies/L, and cytology indicated squamous cell carcinoma. Cervical mucosal biopsy: microscopic examination and pathological diagnosis showed fragmented cervical tissue with scant, fragmented, and free-lying atypical squamous epithelial tissue, making it difficult to definitively assess stromal invasion, consistent with CIN3 (**Figure 2**). Immunohistochemistry results were as follows: P16 positive, P40 positive, Ki67 positive in all layers, PDL-1D with low expression (approximately 5%), and MSS (**Table 1**).

**Table 1:** Timeline of the clinical course, key investigations, and management.

Time	Key events (investigations and management)
Day −5	Onset of progressively worsening dyspnea and right chest pain; low-grade fever.
Day 0	Emergency visit; SARS-CoV-2 nucleic acid test positive. Chest CT suggested right lower lobe pneumonia and possible PE.
Day 0–2	Empirical moxifloxacin at the local hospital for suspected bacterial co-infection; no clinical improvement.
Day 1	CTPA confirmed PE in the right lower lobe. Echocardiography revealed multiple mobile masses in the right atrium/ventricle and tricuspid apparatus.
Day 1–10	Hospital admission; therapeutic UFH with APTT target 60–80 s. Repeat echocardiography showed persistent/enlarging right-sided intracardiac mass.
Day 10–14	Vaginal bleeding during anticoagulation prompted gynecologic evaluation (transvaginal ultrasound, pelvic MRI). PET-CT, HPV testing, and cervical biopsy supported advanced cervical SCC with metastatic disease and suspected tumor thrombus. MDT discussion; high surgical risk; patient/family chose conservative management.
Follow-up	Best supportive care with individualized risk–benefit decisions for anticoagulation given persistent PE/tumor-associated thrombosis and bleeding risk. Survival: 14 months after diagnosis.

To further clarify the disease staging, treatment options, and prognosis, a multi-disciplinary team (MDT) consultation was arranged for the patient involving Oncology, Gynecology, Cardiac Surgery, Pulmonology, and the Intensive Care Unit (ICU). After the systematic discussion, the patient was diagnosed with cervical cancer with pelvic dissemination, along with the presence of both thrombus and tumor thrombus in the right atrium and pulmonary artery. Systemic anticancer therapy or surgical resection of the masses involving the right ventricle and pulmonary artery was deemed to carry an extremely high risk. After comprehensive communication of the patient’s condition and prognosis with the family, they opted for conservative management (**Table 1**).

## Discussion

This case represents a 53-year-old woman with cervical cancer who developed synchronous cardiac and pulmonary metastasis along with pulmonary embolism, following COVID-19 infection. The uniqueness of this report lies in the concurrent diagnosis of cervical cancer, cardiac tumor thrombi, and pulmonary embolism in a patient initially admitted for respiratory symptoms, highlighting the diagnostic challenge posed by overlapping presentations of infectious, thrombotic, and neoplastic diseases. The multidisciplinary approach employed in this case underscores the complexity of managing rare metastatic patterns in gynecological malignancies.

Cardiac metastasis from cervical cancer is exceptionally rare, with autopsy studies suggesting an incidence of approximately 2.3% among metastatic tumors. Tumors below the diaphragm seldom metastasize to the heart due to the cardiac dynamic motion, rapid blood flow, and limited lymphatic communication with pelvic structures (4). In this case, the likely route of spread was hematogenous via the cervical venous plexus through the inferior vena cava to the right heart, along with tumor cell filtration in the lungs. Clinically, such patients often present with dyspnea, chest pain, or signs of pulmonary embolism, which can obscure the underlying malignancy. Cardiac masses may be misidentified as benign thrombi, particularly in the setting of recent infection or immobility, leading to delayed diagnosis and inappropriate management. In this case, the patient experienced an increase in the cardiac mass after a 10-day course of empirical anticoagulation therapy, which ruled out the possibility of a right heart thrombus. This case illustrates how a cardiac mass detected on echocardiography in a patient with pulmonary embolism should prompt investigation for an occult malignancy, even in the absence of typical gynecologic symptoms.

The prognosis cardiac metastasis is poor, with median survival often less than 6 months (6). Early diagnosis is critical, as advanced disease may preclude curative treatment. In this patient, the simultaneous detection of cervical cancer and cardiac involvement at presentation allowed for rapid multidisciplinary assessment, though the extent of disease and high surgical risk limited therapeutic options. Anticoagulation therapy, initiated for presumed pulmonary embolism, precipitated significant vaginal bleeding, further complicating management. This highlights the need for comprehensive staging, including cardiac evaluation, in patients with advanced cervical cancer, especially when respiratory or cardiovascular symptoms emerge. After the acute bleeding rapid, the patient was given oral anticoagulant to treat PE.

The potential pathological mechanism underlying the pulmonary thrombosis is multifaceted. The confirmed presence of SARS-CoV-2 in endothelial cells suggests that both direct viral effects and indirect effects from perivascular inflammation and coagulopathy play roles in the development of pulmonary vasculopathy in COVID-19. This endothelial dysfunction, combined with systemic inflammation and alterations in the coagulation cascade, creates a prothrombotic state that can persist even after viral clearance. In our patient with occult cervical cancer, this hyperinflammatory state likely exacerbated tumor-associated inflammation, potentially accelerating the progression and metastasis of the underlying malignancy to heart. The failure of the right ventricular mass to regress with anticoagulation alone supports the diagnosis of tumor thrombus rather than simple thrombus. This case illustrates how an acute viral infection can unmask an occult malignancy and precipitate catastrophic thromboembolic events through synergistic inflammatory and prothrombotic mechanisms.

This case underscores the diagnostic challenge of cardiac metastasis in cervical cancer, a rare but aggressive manifestation that often mimics thromboembolic disease. A high index of suspicion is warranted when patients with known or suspected malignancy present with unexplained cardiorespiratory symptoms. Multidisciplinary collaboration is essential to guide appropriate imaging and biopsy strategies. While treatment remains individualized and often palliative due to the high risk of intervention, early detection may allow for aggressive local or systemic therapy in selected cases, potentially improving outcomes. Future reporting of similar cases will help refine diagnostic pathways and therapeutic approaches for this uncommon but clinically significant complication of cervical cancer.

## Data Availability

The raw data supporting the conclusions of this article will be made available by the authors, without undue reservation.
